# Progress in Medicine

**Published:** 1898-09-03

**Authors:** 


### Progress in Medicine.

### FEVERS.

(*Continued from page* 371.)

**Tetanus.**—A valuable *résumé* of recent work on this disease is given by by Councilman and Mallory.[29] Blumenthal thinks there are two poisons at work, one the simple product of the bacillus and the other produced by the union of the former with the protoplasm of the cells. The latter is unaffected by anti-toxin when once formed. Ransom[30] finds that the poison cannot enter through the intact mucous membrane of the intestinal tract, but is discharged unchanged in the fæces. Roux and Borrell[31] show that intracranial injections of anti-toxin are much more efficacious for many animals than subcutaneous ones. Metschnikoff[32] does not believe that immunity depends on anti-toxic power. The latter does not exist in creatures below the mammalian kingdom. The alligator indeed possesses it in a high degree, but neither do fishes show it, nor invertebrates, though immune at their normal temperatures. Tizzoni[33] reports that immunity against the tetanus bacillus can be produced by injections of Fraenkel's pneumococcus, which also increases the resistance against tetano-toxin. The immunity very rapidly takes place, and is even found when the two bacilli are injected at the same time. Another theory is that the anti-toxin of tetanus consists of the dissolved cells of the brain of the animal infected, and that the poison can be neutralised by an emulsion of normal brain substance. Wasserman and Takaki[34] show that such an emulsion does neutralise the poison, immunising and even curing animals who are attacked by it. They believe that the original toxine produced by the bacillus has a special affinity for certain cells of the brain, and when these are injected as an emulsion into the blood they combine with the poison directly and shield the central nervous system. Metschnikoff argues that this is not an anti-toxic action, but that the injections of the emulsion cause increased leucocytosis, by which means the bacilli are destroyed. Schubert[35] relates two unsuccessful cases of treatment by Behring's serum. The injury in the first case was in the finger. Symptoms appeared on the fifth day, injections were made on the sixth, but death took place on the seventh day. In the other patient a wound of the foot was followed by symptoms on the seventh day, when also the serum was given, but death occurred on the tenth. Curnow, at Greenwich,[36] used the anti-toxin from the British Institute for a wound of the thumb on the seventh day, the first symptoms having appeared on the fourth day. Greenwood[37] reports a recovery where length of incubation was unknown, but treatment was commenced on the fourth day of the symptoms with Tizzoni's anti-toxin, followed by that of the British Institute. Fifty c.c. were injected in one day, and a formalin dressing employed. He reports also a fatal case, where no anti-toxin was used, and the symptoms began on the sixth day; also a fatal one where the incubation was uncertain, and the injections were apparently begun on the third day. W. N. Clemmey[38] describes an instance of recovery where the symptoms were delayed till the eleventh day. Amputation and nerve sedatives were employed. Symptoms increased for four days after amputation, which rather bears out the idea that a second poison is formed from that already circulating in the blood. Patteson, of Dublin, had[39] two cases of recovery under anti-toxin, but the symptoms were delayed till about the eleventh day in each instance. Several cases were reported in the Royal Academy of Medicine.[40] McCausland related an instance where the symptoms appeared on the second day. Anti-toxin, excision, and bromides were given and recovery followed. In the other cases no dates are given, except that Stoker referred to an old case of the acute type which recovered under mercury and nitrite of amyl. A. E. Maylard[41] had a patient suffering from cephalic hydrophobic tetanus, where the symptoms appeared on the fifth day after a head injury. Treatment was by chloral, and death occurred on the eighteenth day. The spasms were almost entirely limited to the head. In a recent number Lambert's analysis of forty-seven acute cases treated by anti-toxin was referred to, and the reduction of mortality in them was shown to be 18 per cent. over that usual under older methods. In none of the present cases have we seen a reference to the uselessness of ordinary disinfectants such as carbolic and perchloride against the bacillus unless mixed with a half per cent. hydrochloric, or caustic potash, or trichloride of iodine.

**Rabies.**—Professor F. J. Allen[42] gives an interesting account of his personal experiences at the Pasteur Institute, Paris, after being bitten in the hand by a stray dog. He notices the disinfection of the needle of the syringe was carried out before it is introduced into a fresh patient, but not afterwards, which he suggests may allow contamination of the syringe itself. There is some pain for two or three hours after the injections, especially on the first few days, as well as great lassitude, but no headache or digestive disturbances. The total mortality has been only ·48 per cent. in 18,645 cases treated, if we exclude those who arrived too late, so that symptoms appeared within 15 days of the injections.

**Snake Poisons.**—Stephens and Myers[43] have investigated the action of anti-toxins as shown in the effects of cobra poison. It has been argued that the cells of an animal body are needed for the action of an anti-toxin, while others hold that the toxin and its antidote react chemically without the aid of the living body. Now, the cobra poison, when added to blood, prevents coagulation in a test tube, but if it is previously mixed with sufficient anti-venomous serum coagulation takes place normally. With various precautions the authors found that one mgrm. of cobra poison lost its power of hæmolysis by mixture with one c.cm. of serum; and, further, that this amount of poison, the minimum lethal dose for a guinea-pig, was innocuous if thus mixed beforehand. The chemical reaction, therefore, is the cause of the immunity. If larger doses are given this law does not hold good, which they explain by the supposed presence of two poisons; the second, though a weaker one, is not neutralised by the anti-toxin. W. Myers[44] thinks it is a nerve poison which acts on the respiratory centre. He has endeavoured to test Wasserman's theory that immunity is produced by neutralisation of the poison with the substance of certain cells in the body for which it has an affinity. He tried emulsions of various organs as an antidote to cobra poison, but found only one, the cortex of the supra-renal bodies, which can neutralise the poison, and that only in minimal doses. He considers it is not a true anti-toxin, but that, like bile, it somehow raises the resistance of the cells of the body.

Calmette's serum has proved to be really efficient as a cure in several instances. He claims[45] that not one case which has received an injection of serum has failed out of a great number of observations. He has distributed the remedy in all parts of the world during the last two years. Ten c.cm. are injected within two, or at least four, hours after a bite, and its strength is such that the horses from whom it is obtained can bear an injection of a gramme of dry venom at once without inconvenience. Moreover, he seems to have proved that the toxic principle of the venoms of different kinds of snakes is identical, and that the serum is efficacious against all, though the vulnerability of animals varies, and the poisons themselves have minor diversities in their mode of action.

**Yellow Fever.**—Of late the outbreaks of this disease have created terrible excitement, not only in the southern districts of the United States, but also on account of its ravages in the Cuban expedition. Exposure to the sun, intemperance, and bad hygienic conditions may predispose towards infection, but in certain districts the disease is endemic. Izett Anderson, in a work on "Yellow Fever," recommends large initial doses of calomel and quinine, followed by a mixture of carbolic acid and bicarbonate of potash, dry cupping over the loins, nitrous ether, and wet packs, but the mortality is very great. Sanarelli[46] announces that his serum acts only against the microbes, and not against the toxins, and hence is only useful in early stages. In eight cases treated at Rio only one recovered out of those treated on the fourth day of the fever, while three treated at an early stage were saved. At San Carlos twenty-two cases showed only five deaths, and Sanarelli found that large doses injected intravenously gave the best results. It should be stated that the average mortality in the capital of Brazil is about 50 per cent. As a preventative Sanarelli injected all the inhabitants of a certain prison where an outbreak occurred, and entirely stopped it at once.

**The Plague.**—The riots at Bombay led to the abolition of domciliary visits, and the outbreak reached the number of 1,800 deaths in one week alone, in spite of every available effort at prevention and treatment.[47] In Poonah the epidemic soon came to an end, but part of the Nizam's dominions were infected. At Jeddah, also, an outbreak has taken place, which renders the danger of an extension to Europe imminent.[48] Though it seems impossible to prevent the annual pilgrimage to Meccah, *viâ* Jeddah, measures have been at once taken to prevent the return of pilgrims to Egypt, except under severe restrictions. By May, however, Bombay began to find the epidemic decreasing, but cases were reported from Calcutta.[49] Inoculations are being used on a large scale with some degree of success, but the mortality of unvaccinated natives reaches 75 or 80 per cent. of those attacked.

[29] Amer. Jour. Med. Sci., July. [30] Deutsche Med. Woch., Feb. 24. [31] Annales de l'In. Pasteur, April. [32] Amer. Jour. Med. Sci., July. [33] Brit. Med Jour., June 18. [34] Clin. Jour., Mar. 16. [35] Brit. Med. Jour., April 16. [36] Lancet, April 30. [37] Ibid. [38] Lancet, May 28. [39] Dublin Jour. Med. Sci., Feb. [40] Ibid. [41] Glasgow Med. Jour., June. [42] Birmingham Med. Rev., Jan. [43] Brit. Med. Jour., Mar. 5. [44] Lancet, July 2. [45] Brit. Med. Jour., May 14. [46] Brit. Med. Jour., April 16, [47] Brit. Med. Jour., Mar. 26. [48] Brit. Med. Jour., April 2. [49] Lancet. May 2.

